# A new home for microbes

**DOI:** 10.7554/eLife.48493

**Published:** 2019-06-21

**Authors:** Raphael Eisenhofer, Alan Cooper

**Affiliations:** 1 Australian Centre for Ancient DNAUniversity of AdelaideAdelaideAustralia

**Keywords:** fossils, microbiome, proteins, Other

## Abstract

Modern microorganisms growing in fossils provide major challenges for researchers trying to detect ancient molecules in the same fossils.

**Related research article** Saitta ET, Liang R, Lau MCY, Brown CM, Longrich NR, Kaye TG, Novak BJ, Salzberg SL, Norell MA, Abbott GD, Dickinson MR, Vinther J, Bull ID, Brooker RA, Martin P, Donohoe P, Knowles TDJ, Penkman KEH, Onstott T. 2019. Cretaceous dinosaur bone contains recent organic material and provides an environment conducive to microbial communities. *eLife* **8**:e46205. doi: 10.7554/eLife.46205

In the 1993 box office hit *Jurassic Park*, scientists ‘resurrect’ dinosaurs by cloning dinosaur DNA that had somehow been preserved in amber for over 65 million years. While *Jurassic Park* was a commercial success and kindled public interest in bringing woolly mammoths and other extinct animals back to life, there is no convincing evidence to suggest that it might be possible to bring back species that became extinct even relatively recently.

It is well known that recent sub-fossils (material that has not yet turned to rock) can contain preserved DNA and proteins such as collagen. However, recent work has shown even true fossils contain organic material that can shed light on how extinct organisms may have looked or behaved in the past. For example, molecules that cause pigmentation (e.g. melanin and porphyrins) have been found in fossils that are hundreds of millions of years old (Zhang et al., 2010).

There have been numerous high-profile claims of DNA and proteins surviving over geological periods of time. But degradation rates suggest that DNA should not survive much longer than ~700,000 years, the age of the oldest genome sequence to be retrieved so far from a preserved bone (Orlando et al., 2013). While protein degradation rates are less clear, the oldest validated proteins to be retrieved are around 4 million years old (Demarchi et al., 2016).

A key challenge is that the reaction used to detect preserved DNA, known as PCR amplification, can easily be contaminated with modern molecules that outcompete the trace-level targets being amplified. As a result, the early stages of research into ancient DNA were characterised by spectacular reports of multi-million-year-old DNA (from plant fossils, and then amber) that could not be replicated. Accordingly, a set of criteria for testing ancient DNA results was established, involving multiple controls and built around the null hypothesis that contamination must be assumed to be present in any result (see, for example, Cooper and Poinar, 2000). Similar criteria are now being adopted in other fields that involve searching for trace-level biomolecules, ranging from proteins in ancient remains to microbial DNA in samples of human placenta in modern biological studies (Hendy et al., 2018; Eisenhofer et al., 2019; Bushman, 2019).

While there have been a series of publications claiming to have identified authentic biomolecules from dinosaur remains – including DNA, collagen, bone cells, and blood vessels – these studies have been characterised as having few controls and lacking robust methodology (Wang et al., 1997; Buckley et al., 2008). Now, in eLife, Evan Saitta of the Field Museum of Natural History in Chicago and co-workers based at institutes in China, Canada, the United Kingdom and the United States report a rigorous examination of the organic molecules preserved within freshly evacuated dinosaur bones that are somewhere between 66 and 100 million years old (Saitta et al., 2019).

Saitta et al. used a veritable arsenal of experimental techniques to demonstrate that no original ancient proteins could be detected within the dinosaur bones. Instead, they found that sub-surface fossils were comprehensively infiltrated by modern molecules, resulting in a range of genetic and biochemical signals that were comparatively different to those in the surrounding rock. To investigate if the signals resulted from micro-organisms, Saitta et al. focused their efforts on the most abundant life forms on earth – bacteria. They observed that the concentration of bacterial DNA was 50 times higher inside the bone compared to the surrounding rock. Further genetic analyses using the 16S rRNA gene (a method commonly used to identify bacteria) revealed that the microbial community localised inside the dinosaur bone was different. This led Saitta et al. to hypothesise that microbes could be using the bone as a habitat or even a source of food.

Previous studies of dinosaur bone have reported the detection of fossil soft tissues, such as blood vessels and bone cells. However, the discovery of microbial habitats inside the bone raises the possibility that these apparent fossil soft tissues might actually represent artefacts caused by microbial biofilms (communities of bacteria stuck to the bone surface). Microorganisms are also likely to compromise the survival and identification of genuine ancient biomolecules by influencing the chemical composition and breakdown of fossil bone. Some fungi can even produce proteins such as collagen (Celerin et al., 1996), which potentially undermines previous studies that have used collagen antibodies.

Current models of biomolecular degradation suggest it should not be possible for dinosaur-era DNA and proteins to survive until the present, and finding that fossil bones can be infiltrated with microorganisms makes this increasingly unlikely. From ancient DNA and proteins millions of years old to modern microbes in supposedly sterile body-sites, history has clearly shown us that it is all too easy for highly sensitive molecular techniques to generate questionable results. The central issue remains how possible it is to both authenticate and falsify the results, which is why we need robust experimental guidelines (Cooper and Poinar, 2000; Hendy et al., 2018; Eisenhofer et al., 2019) to keep science separate from fiction.
